# Evaluating the Clinical Decision-Making Ability of Large Language Models Using MKSAP-19 Cardiology Questions

**DOI:** 10.1016/j.jacadv.2023.100658

**Published:** 2023-10-26

**Authors:** Paul C. Lee, Samin K. Sharma, Shreya Motaganahalli, Andy Huang

The rise of large language models (LLMs) has brought about a paradigm shift in artificial intelligence, with significant potential for their application in medicine. These LLMs employ deep learning techniques to process and generate human-like text based on massive data sets, enabling them to “understand” context and perform complex tasks with remarkable accuracy. LLMs can transform the way practitioners access medical information, support clinical decision-making, and interact with patients.^1^ We tested 2 state-of-the-art LLMs on 120 cardiology questions from the Medical Knowledge Self-Assessment Program (MKSAP-19)^2^: 1) OpenAI’s ChatGPT-3.5, trained on extensive web-crawled text, and its updated version, ChatGPT-4^3^; and 2) Stanford Center for Research on Foundation Models’ PubMedGPT,^4^ an LLM based on GPT-2 architecture, trained on biomedical literature, achieving 50.3% accuracy on MedQA-USMLE.^5^

**Figure 1:**
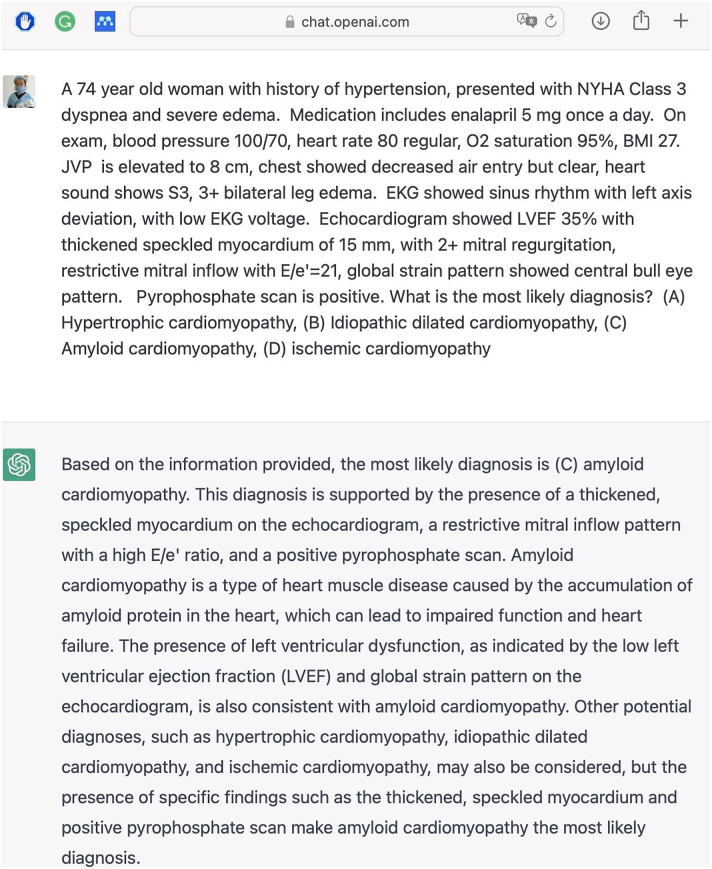
**Example of ChatGPT 3.5 Answering a Test Question in the Style of MKSAP-19 (Due to Copyright Restrictions, This Is Not an Actual Question From MKSAP-19)** MKSAP-19 = Medical Knowledge Self-Assessment Program.

We evaluated 2 LLMs and physicians using MKSAP-19's cardiology set of 120 clinical case questions, accessible to subscribers only and not crawlable. The publisher set the passing score at 50%. We input questions verbatim into the LLMs and annotated the responses for correct answers (Figure 1, which shows an example similar to MKSAP-19 questions but not an actual question due to copyright). The average performance of physician users was from the MKSAP-19 website. We conducted pairwise comparisons using chi-squared tests with Bonferroni correction, setting the adjusted significance level at α = 0.05/4 = 0.0125, using Stata 17.0. The pairwise comparisons included ChatGPT-3.5 vs physicians, PubMedGPT vs physicians, ChatGPT-4 vs physicians, and ChatGPT-3.5 vs PubMedGPT.

ChatGPT 3.5 met the passing criteria, answering 66 questions correctly (55%), numerically but not statistically lower than physicians (60%, *P* = 0.43). PubMedGPT fell short, scoring 27%, significantly lower than physicians (*P* < 0.001) and ChatGPT-3.5 (*P* < 0.001). ChatGPT-4 outperformed average MKSAP-19 users (80% vs 60%, *P* = 0.0004), matching the first author, a seasoned cardiologist (80%). The performance comparison highlights the different capabilities of the LLMs, with ChatGPT-3.5 and ChatGPT-4 demonstrating a level of competence that approaches that of physicians, while PubMedGPT struggles to meet the passing threshold.

The performance of ChatGPT 3.5 and the ChatGPT 4.0 in passing the MKSAP-19 cardiology section demonstrates progress for medical LLMs, despite limitations: 1) overconfidence, where the model expresses certainty when uncertainty should be acknowledged; 2) incorrect numerical computations, specifically for calculation of Atherosclerotic Cardiovascular Disease risk scores; 3) inability to interpret images; and 4) hallucination, where the model generates information not based on factual data. In contrast, PubMedGPT performed poorly, potentially due to the difference in neural network parameters (ChatGPT: 175 billion, PubMedGPT: 2.7 billion).

One limitation of this study is that exam questions may not reflect real-life clinical challenges. Nonetheless, GPT-4's impressive performance on the MKSAP-19 cardiology questions indicates its potential ability to process medical information, understand complex medical contexts, and provide evidence-based recommendations. This ability could support health care professionals in making informed decisions, streamlining clinical workflows, and improving patient outcomes by automating complex tasks and contributing to the development of personalized treatment plans. We have designed a set of ChatGPT prompts that cardiologists can readily utilize for various applications–including tasks such as ranking the probability of side effects (eg, QT prolongation) given a medication list or facilitating curb-side cardiology consults–that is available upon request.

In addition to helping clinical workflow, LLM advancements could revolutionize cardiology research by analyzing literature more efficiently, generating hypotheses that incorporate interdisciplinary insights, optimizing clinical trials by refining participant selection, and improving personalized treatment by rapidly synthesizing individual patient data with the latest research findings.

In just 1 year, LLMs have progressed from barely passing medical student licensing exams, as demonstrated by PubMedGPT's performance on the United States Medical Licensing Examination,^5^ to achieving performance on par with seasoned cardiologists in a particular context (MKSAP-19 cardiology questions), as shown by GPT-4 in our study. The swift advancements in LLMs highlight their potential as invaluable assistants in cardiology practice. Integrating these models into clinical settings has the potential to enhance efficiency, streamline workflows, and ultimately contribute to improved patient outcomes. However, it is crucial to remain vigilant about their limitations and to continue refining their capabilities to ensure their safe and effective use in medical practice.
